# Development of a Highly Sensitive Loop-Mediated Isothermal Amplification (LAMP) Method for the Detection of *Loa loa*

**DOI:** 10.1371/journal.pone.0094664

**Published:** 2014-04-10

**Authors:** Pedro Fernández-Soto, Prosper Obolo Mvoulouga, Jean Paul Akue, Julio López Abán, Belén Vicente Santiago, Miguel Cordero Sánchez, Antonio Muro

**Affiliations:** 1 IBSAL-CIETUS (Instituto de Investigación Biomédica de Salamanca-Centro de Investigación de Enfermedades Tropicales de la Universidad de Salamanca), Facultad de Farmacia, Universidad de Salamanca, Salamanca, Spain; 2 Deparment of Medical Parasitology, Centre International de Recherches Medicales de Franceville (CIRMF), Franceville, Gabon; 3 Department of Infectious Diseases, Service of Internal Medicine, University Hospital of Salamanca, Salamanca, Spain; New England Biolabs, Inc., United States of America

## Abstract

The filarial parasite *Loa loa*, the causative agent of loiasis, is endemic in Central and Western Africa infecting 3–13 million people. *L. loa* has been associated with fatal encephalopathic reactions in high *Loa*-infected individuals receiving ivermectin during mass drug administration programs for the control of onchocerciasis and lymphatic filariasis. In endemic areas, the only diagnostic method routinely used is the microscopic examination of mid-day blood samples by thick blood film. Improved methods for detection of *L. loa* are needed in endemic regions with limited resources, where delayed diagnosis results in high mortality. We have investigated the use of a loop-mediated isothermal amplification (LAMP) assay to facilitate rapid, inexpensive, molecular diagnosis of loiasis. Primers for LAMP were designed from a species-specific repetitive DNA sequence from *L. loa* retrieved from GenBank. Genomic DNA of a *L. loa* adult worm was used to optimize the LAMP conditions using a thermocycler or a conventional heating block. Amplification of DNA in the LAMP mixture was visually inspected for turbidity as well as addition of fluorescent dye. LAMP specificity was evaluated using DNA from other parasites; sensitivity was evaluated using DNA from *L. loa* 10-fold serially diluted. Simulated human blood samples spiked with DNA from *L. loa* were also tested for sensitivity. Upon addition of fluorescent dye, all positive reactions turned green while the negative controls remained orange under ambient light. After electrophoresis on agarose gels, a ladder of multiple bands of different sizes could be observed in positive samples. The detection limit of the assay was found to be as little as 0.5 ag of *L. loa* genomic DNA when using a heating block. We have designed, for the first time, a highly sensitive LAMP assay for the detection of *L. loa* which is potentially adaptable for field diagnosis and disease surveillance in loiasis-endemic areas.

## Introduction

Loiasis is a neglected tropical disease caused by the nematode *Loa loa*, an endemic filarial parasite transmitted by Tabanid flies of the genus *Chrysops* in the forest and savannah regions of Central and Western Africa infecting between 3 and 13 million people. Although typical clinical presentation of loiasis includes oedema (Calabar swellings), migration of the adult worm through the sub-conjunctiva (“African eyeworm”) and pruritus, infected individuals may exhibit a range of symptoms. On the other hand, indigenous inhabitans of endemic areas are typically clinically asymptomatic even in the setting of high parasite burden [1].

In recent years, *L. loa* has come increasingly to light as it has been associated with severe adverse reactions (including fatal encephalopathies) in high *Loa*-infected individuals receiving ivermectin during mass drug administration programs for the control of onchocerciasis and lymphatic filariasis. This had a negative impact on control of these two diseases in several areas of co-endemicity with loiasis [2], [3]. A greatest risk for *Loa*-related post-ivermectin encephalopathy has been reported to appear with microfilaremia above 8,000 organism/mL [4], [5]. In this context, it is necessary the identification of individuals with high-level *L. loa* microfilaremia in order to prevent the interruption of mass drug administration programs in potentially loiasis endemic areas where ivermectin treatment was initially planned.

In endemic areas, the only diagnostic method routinely used is the microscopic examination of mid-day blood samples by thick blood film. This method requires expert training in parasite morphology and requires a great effort to process large numbers of samples so, in practice, is not suitable for large-scale surveys. Serological assays are an alternative, but suffer from poor specificity [6] and cannot discern between active and prior infection or quantify microfilaremia [7], [8], [9]. Regarding the molecular methods, in recent years both conventional [10], [11], [12], [13], [14] and real-time PCR assays [15] have been developed for *L. loa* DNA detection. While these studies have demonstrated that PCR-based assays provided reliable, specific and sensitive tools, they are not generally available for use in endemic areas because highly skilled personnel and precision thermocyclers are needed. Therefore, the development of cost-effective, simple and rapid detection methods is still needed for the diagnosis of loiasis.

An alternative to PCR is a technique named loop-mediated isothermal amplification (LAMP). This assay is a one-step amplification reaction that amplifies a target DNA with high specificity, efficiency and rapidly under isothermal conditions [16]. LAMP employs a DNA polymerase (*Bst* polymerase) with strand-displacement activity, along with two inner primers (FIP, BIP) an outer primers (F3, B3) which recognize six separate regions within a target DNA. Additionally, the LAMP assay does not require an expensive thermocycler and allows simple visual detection of products [17], [18]. It has been applied successfully to detect several pathogenic parasites, including filarial parasites, such as *Dirofilaria immitis* [19], *Brugia* spp. [20] and *Wuchereria bancrofti* [21].

The objective of this work was to design, for the first time, a LAMP-based approach for the highly sensitive detection of *L. loa* DNA potentially adaptable for field diagnosis of loiasis.

## Materials and Methods

### Ethics statement

The *Loa loa* adult worm used as source of DNA in our study was obtained by surgical removal from the eye of a Gabonese immigrant patient infected with the parasite as part of public health diagnostic activities at Tropical Medicine Consultation of University Hospital of Salamanca, Spain (Protocol no. 22.832). Then, sample was preserved in 70% ethanol. Patient was given detailed explanations about the aims, procedures and possible benefit of the study. Written informed consent was obtained from patient and sample was coded. Dr. Miguel Cordero (Specialist Head of Tropical Medicine Consultation) had contact with the patient and also had access to the patient's records.

The human blood samples used in this study were obtained from healthy blood donors (laboratory staff); participation was voluntary and all participants were given detailed explanations about the aims, procedures and possible benefit of the study. Written informed consent was obtained from all subjects and samples were coded and treated anonymously.

### DNA extraction

#### Parasites

*Loa loa* genomic DNA was extracted from an adult worm preserved in 70% ethanol using DNeasy Blood & Tissue Kit (Qiagen, Hilden, Germany) following the manufacturer's instructions. The concentration of *L. loa* DNA adult worm was measured three times by spectrophotometry using a Nanodrop ND-100 spectrophotometer (Nanodrop Technologies) to obtain an average concentration and then diluted with ultrapure water to a final concentration of 2.5 ng/μL. Subsequently, a 10-fold serial dilution was prepared ranging from 1×10^−1^ to 1×10^−13^ ng/μL and stored at −20°C until use. Several helminths, including four nematodes namely *Mansonella perstans*, *Brugia pahangi*, *Strongyloides venezuelensis* and *Anisakis simplex*, seven trematodes namely *Schistosoma mansoni*, *S. haematobium*, *S. intercalatum*, *S. bovis*, *Fasciola hepatica*, *Echinostoma caproni* and *Dicrocoelium dendriticum*, and three cestodes namely *Hymenolepis diminuta*, *Taenia taeniformis* and *Echinococcus granulosus* were included as heterogeneous control samples for assessing the specificity of LAMP assays. All these parasites materials were preserved in 70% ethanol and kept at −20°C until the extraction of DNA. Then, DNA was extracted and measured by the same methods as described for *L. loa* adult worm. In order to look for protein contaminations a common purity check by measuring the A_260_/A_280_ ratio was made for all samples.

#### Blood samples

Whole blood was taken from healthy staff donors, divided into aliquots of 200 μL and then artificially contaminated with genomic DNA from *L. loa* 10-fold serially diluted as prepared above. Then, DNA was extracted using NucleoSpin Blood kit (Macherey-Nagel, Germany) following the manufacturer's instructions.

### *Loa loa* specific LAMP oligonucleotide design

An exclusive 839 base pair (bp) species-specific repetitive DNA sequence from *L. loa* was retrieved from GenBank (Accesion No. M34259.1) [10] and was used for the design of specific primers with Primer Explorer V4 software (Eiken Chemical Co., Ltd., Japan; http://primerexplorer.jp/e/). The primers were designed and selected based on the criteria described in “A Guide to LAMP primer designing” (http://primerexplorer.jp/e/v4_manual/index.html). Specific LAMP primer set selected consisting of primers F3, B3, FIP (F1c+F2) and BIP (B1c+B2) are shown in table 1. All of the primers were of HPLC grade (TIB-MOLBIOL, Germany).

**Table 1:** Sequences of LAMP primers for the amplification of the repetitive DNA sequence from *Loa loa* (GenBank Accesion No. M34259.1).

Primer^a^	Length (bp)	Sequence (5′-3′)
F3	22	TCAGCATTTATAACAAGAAGCA
B3	24	GAATTACTGTGATGGATTACTACA
FIP (F1c+F2)	40	CAGCTCCTCACTGTGGCATG-CCAAAAAACACCGATGGATG
BIP (B1c+B2)	43	TTAAGCGACTTCGTGCTGCTAC-TCAGAAAACAACACTGTATCC

a F3, forward outer primer; B3, reverse outer primer; FIP, forward inner primer (comprising F1c and F2 sequences); BIP, reverse inner primer (comprising B1c and B2 sequences).

#### LAMP F3 and B3 primer specificity tested by PCR

The outer LAMP primer pair, designated F3 and B3 (Table 1), was initially tested for *L. loa* specificity by PCR to verify whether the correct target was amplified. An amplicon of 189 bp was expected for *L. loa*. The PCR assay was conducted in 25 μL reaction mixture containing 2.5 μL of 10x buffer, 1.5 μL of 25 mmol/L MgCl_2_, 2.5 μL of 2.5 mmol/L dNTPs, 0.5 μL of 100 pmol/L F3 and B3, 2 U *Taq*-polymerase and 2 μL of DNA template. Initial denaturation was conducted at 94°C for 1 min, followed by a touchdown program for 15 cycles with successive annealing temperature decrements of 1.0°C every 2 cycles. For these 2 cycles, the reaction was denatured at 94°C for 20 s followed by annealing at 59°C–57°C for 20 s and polymerization at 72°C for 30 s. The subsequent 15 cycles of amplification were similar, except that the annealing temperature was 56°C. The final extension was performed at 72°C for 10 min.

The sensitivity of the PCR was also assayed to establish the detection limit of *L. loa* DNA with 10-fold serial dilutions prepared as mentioned above. The assay was performed with 2 μL of the diluted template in each case. Negative (no DNA template) controls were included. The PCR products (10 μL of each) were subjected to 2% agarose gel electrophoresis, stained with ethidium bromide, and visualized under UV light.

### LAMP assay

The LAMP assay was performed using the *Loopamp DNA amplification Kit* (Eiken Chemicals Co., Tokyo, Japan) following manufacturer's instructions. Briefly, the reaction was carried out with a total of 25 μL reaction mixture containing 12.5 μL of 2x Reaction Mix, 40 pmol of each FIP and BIP primers, 5 pmol of each F3 and B3 primers, 1 μL of *Bst*-DNA polymerase, along with 2 μL of DNA template. To establish the standard protocol for the LAMP method, the reaction mixture was incubated in a conventional heating block (K Dry-Bath) or in a thermocycler (Mastercycler Gradient-96well; Eppendorf) at a range of temperatures (61, 63 and 65°C) for 60 min to optimize the reaction conditions and then heated at 80°C for 5 min to terminate the reaction. The optimal temperature was determined and used in the subsequent tests. Because of the highly sensitivity of LAMP reaction, DNA contamination and carry-over of amplified products were prevented by using sterile tools at all times, performing each step of the analysis in separate work areas, minimizing manipulation of the reaction tubes and closing them with flexible parafilm. Negative controls (ultrapure water) were included in each LAMP reaction. These controls never amplified.

#### Detection of LAMP product

Amplified DNA in the LAMP reaction causes turbidity due to the accumulation of magnesium pyrophosphate, a by-product of the reaction. After inactivation of the reaction tubes, the turbidity of reaction mixture was inspected by the naked eye. The LAMP amplification results were also visually detected by adding 2 μL of 1∶10 diluted 10,000X concentration fluorescent dye SYBR Green I (Invitrogen) to the reaction tubes. A successful LAMP reaction would turn to green; otherwise, it would remain orange. Additionally, the LAMP products were also monitored using 2% agarose gel electrophoresis stained with ethidium bromide, visualized under UV light and then photographed using the ultraviolet (UV) image system (Gel documentation system, UVItec, UK).

#### Specificity and sensitivity of LAMP

To determine the specificity of the LAMP assay for L. loa, DNA samples from other parasites including *Mansonella perstans, Brugia pahangi, Strongyloides venezuelensis Anisakis simplex, Schistosoma mansoni, S. haematobium, S. intercalatum, S. bovis, Fasciola hepatica, Echinostoma caproni, Dicrocoelium dendriticum, Hymenolepis diminuta, Taenia taeniformis* and *Echinococcus granulosus* were tested as controls. The sensitivity of the assay was confirmed using genomic DNA from L. loa which was 10-fold serially diluted as mentioned above and also with the human blood samples spiked with the same dilutions.

## Results

### PCR verification of target length and sensitivity

**Figure 1:**
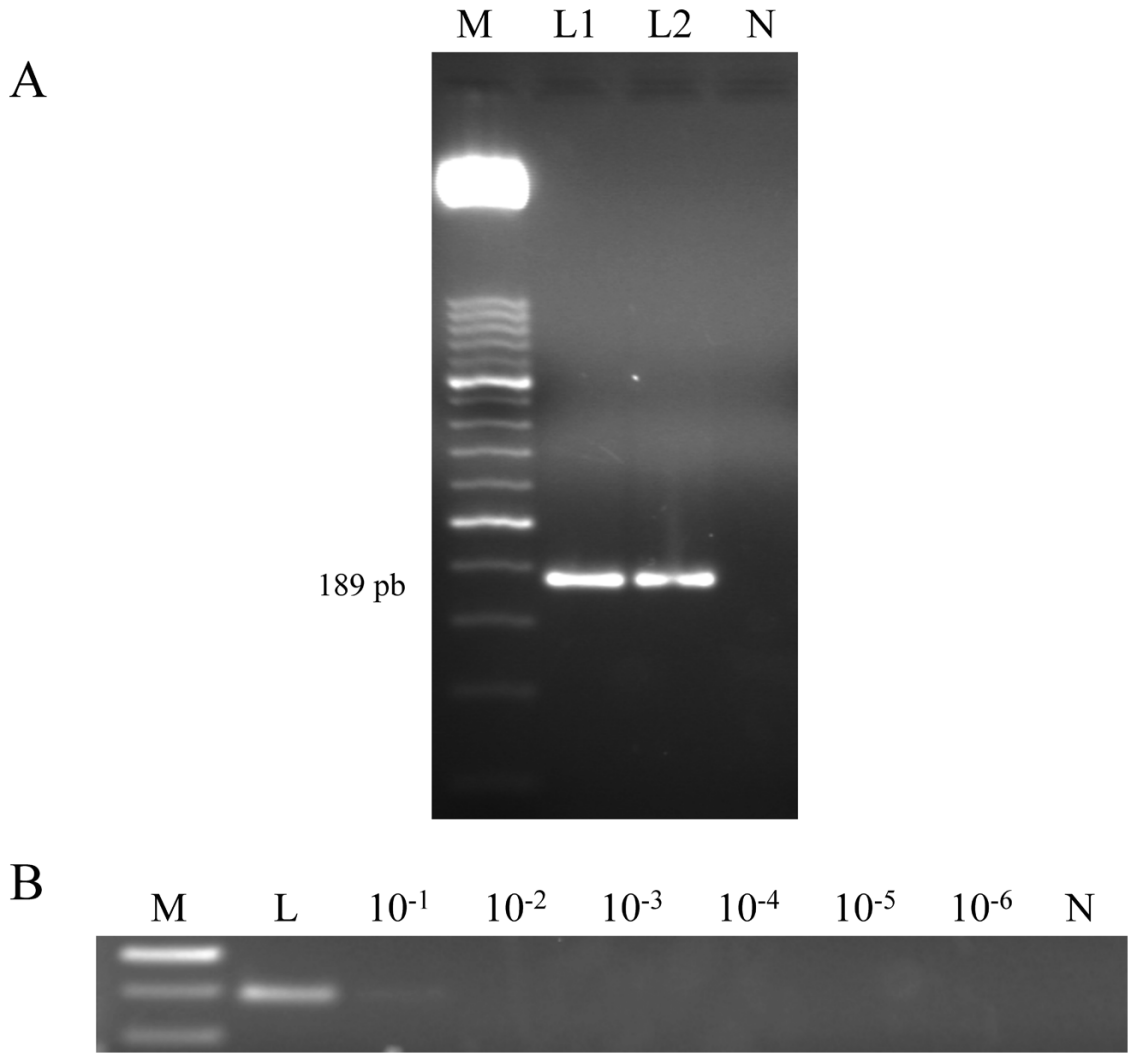
PCR verification and detection limit. (A) PCR verification of expected target length using LAMP outer primers F3 and B3. Lane M, 50 bp DNA ladder (Molecular weight marker XIII, Roche); lanes L1 and L2: *Loa loa* DNA; lane N, negative control (no DNA template). (B) Detection limit of PCR. Lane M, 50 bp DNA ladder (Molecular weight marker XIII, Roche); lane L: *Loa loa* DNA (5 ng); lanes 10^−1^–10^−6^: 10-fold serially dilutions of *L. loa* DNA; lane N, negative control (no DNA template).

The conventional PCR amplification using outer primers F3 and B3 was used to verify whether the correct target was amplified and an expected 189 bp fragment was obtained (Fig. 1A), which is considered to correspond to the 839 bp species-specific repetitive DNA sequence from *L. loa* (GenBank Accesion No. M34259.1) (data not shown). To determine the detection limit of the PCR, a 10-fold serial dilution ranging from 10^−1^ to 10^−6^ of *L. loa* adult worm DNA was amplified. The minimum amount of DNA detectable by PCR was 0.5 ng (Fig. 1B).

### Detection of LAMP product

**Figure 2:**
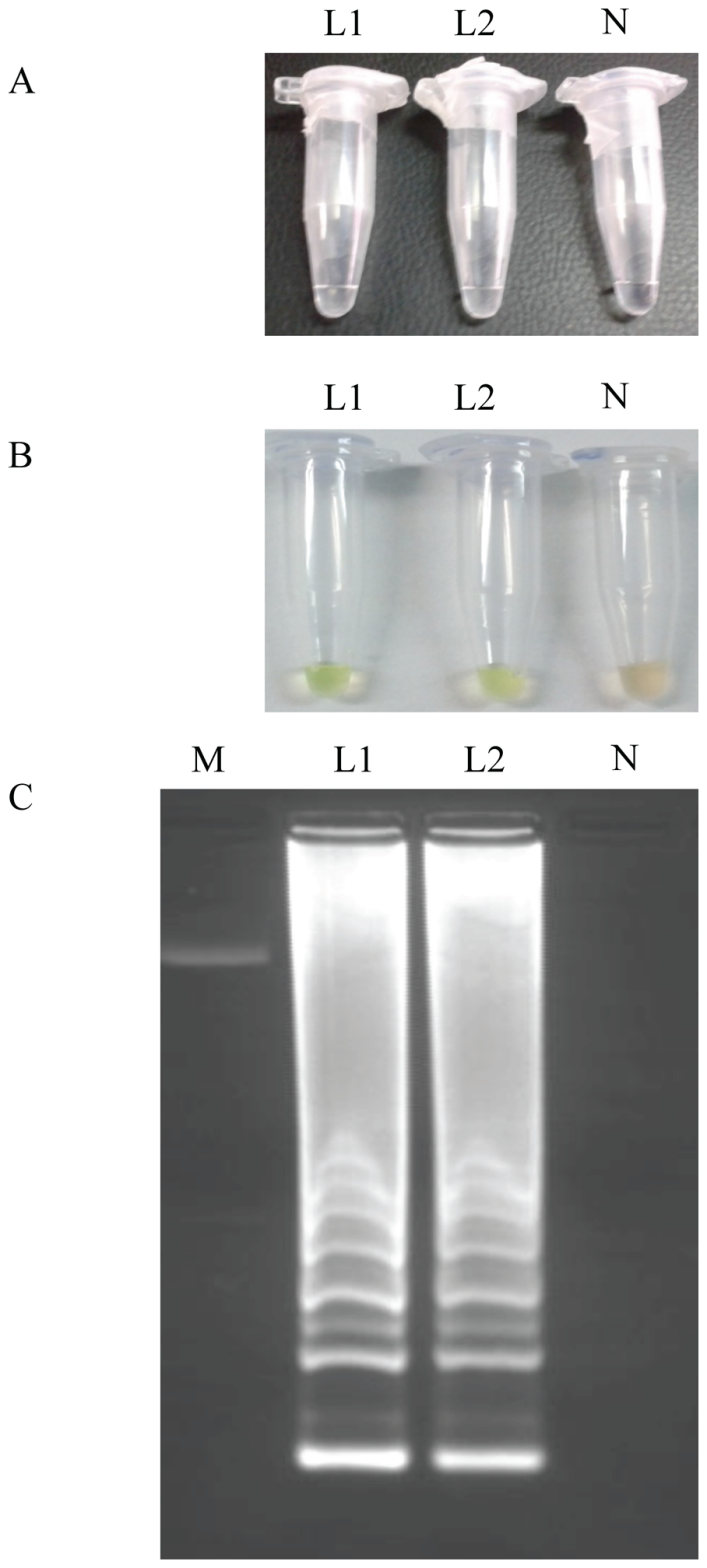
LAMP detection of *Loa loa* genomic DNA samples. (A) By the naked eye; (B) by adding SYBR Green I; (C) on agarose gel stained with ethidium bromide. Lanes L1 and L2: *Loa loa* adult worm genomic DNA; lane N: no template (negative control); lane M: 50 bp DNA ladder (Molecular weight marker XIII, Roche).

The optimum incubation temperature for LAMP assay with the *L. loa* primer set was established using a range of temperatures (61, 63 and 65°C) for 60 min to optimize the reaction conditions and then heated at 80°C for 5 min to terminate the reaction. The LAMP assay could successfully take place at temperatures of 61°C, 63°C and 65°C. However, better results on agarose gels were obtained when using 63°C in both thermocycler and the conventional heating block (data not shown). Thus, the optimum temperature and time for LAMP were choose to be 63°C and 60 min, respectively, and used for all the following applications. Positive LAMP reactions turned turbid whereas negative ones remained clear (Fig. 2A). The original orange color of the SYBR Green I changed to green in case of positive amplification, whereas the original orange color was retained for the negative control with no amplification (Fig. 2B). After electrophoresis on agarose gels a ladder of multiple bands of different sizes could be observed in positive samples (Fig. 2C).

### Specificity of the LAMP assay

**Figure 3:**
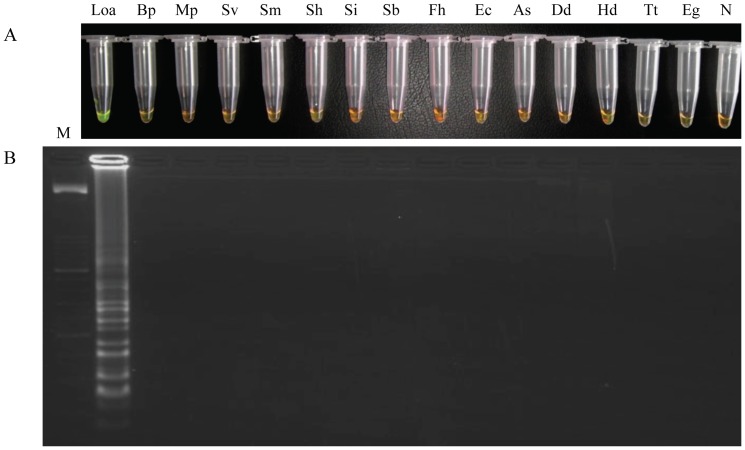
Specificity assessment of LAMP assay. (A) Visual examination of LAMP products by adding SYBR Green I. (B) Agarose gel electrophoresis of amplified products. Lanes Loa, Bp, Mp, Sv, Sm, Sh, Si, Sb, Fh, Ec, As, Dd, Hd, Tt, Eg: DNA from *Loa loa*, *Brugia pahangi, Mansonella perstans, Strongyloides venezuelensis, Schistosoma mansoni, Schistosoma haematobium, Schistosoma intercalatum, Schistosoma bovis*, *Fasciola hepatica*, *Echinostoma caproni, Anisakis simplex*, *Dicrocoelium dendriticum, Hymenolepis diminuta*, *Taenia taeniformis* and *Echinococcus granulosus*, respectively. Lane M: 50 bp DNA ladder (Molecular weight marker XIII, Roche); lane N: negative control (no DNA template).

The specificity of the LAMP assay was tested using heterogeneous DNA samples as controls. Positive amplification was only observed using DNA from *L. loa*. By contrast, DNA samples from other specimens were not amplified. This result indicates that no false-positive amplifications were observed with these heterogeneous species in the LAMP assay (Fig. 3).

### Sensitivity of the LAMP assay

**Figure 4:**
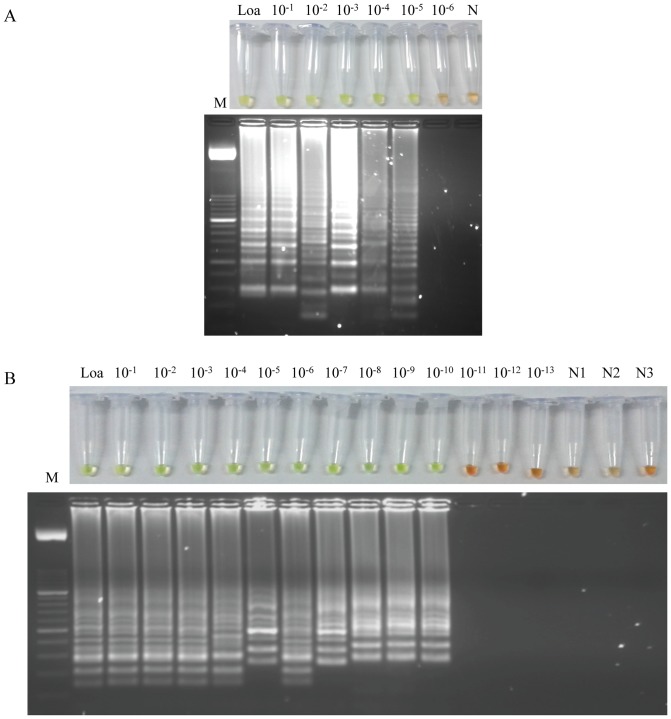
Sensitivity assessment of the LAMP assay for *Loa loa* using serial dilutions of genomic DNA by the addition of SYBR Green I or by visualization on agarose gel stained with ethidium bromide. (A) Sensitivity assessment performed with a thermocycler. (B) Sensitivity assessment performed with a heating block. Lane Loa: genomic DNA from *Loa loa* (5 ng); lanes 10^−1^–10^−13^: 10-fold serially dilutions; lanes N, N1, N2, N3: negative controls (no DNA template). Lane M: 50 bp DNA ladder (Molecular weight marker XIII, Roche).

To determine the limit of detection of the LAMP assay, a 10-fold serial dilution of *L. loa* genomic DNA was amplified by LAMP. The reaction mixtures were incubated in both thermocycler and conventional heating block to compare results. The limit of detection of LAMP using a thermocycler was 50 fg (Fig. 4A), whereas the limit of detection using a conventional heating block was established in 0.5 ag (Fig. 4B), showing that LAMP assay is much more sensitive when a heating block was used to incubate the reaction tubes.

**Figure 5:**
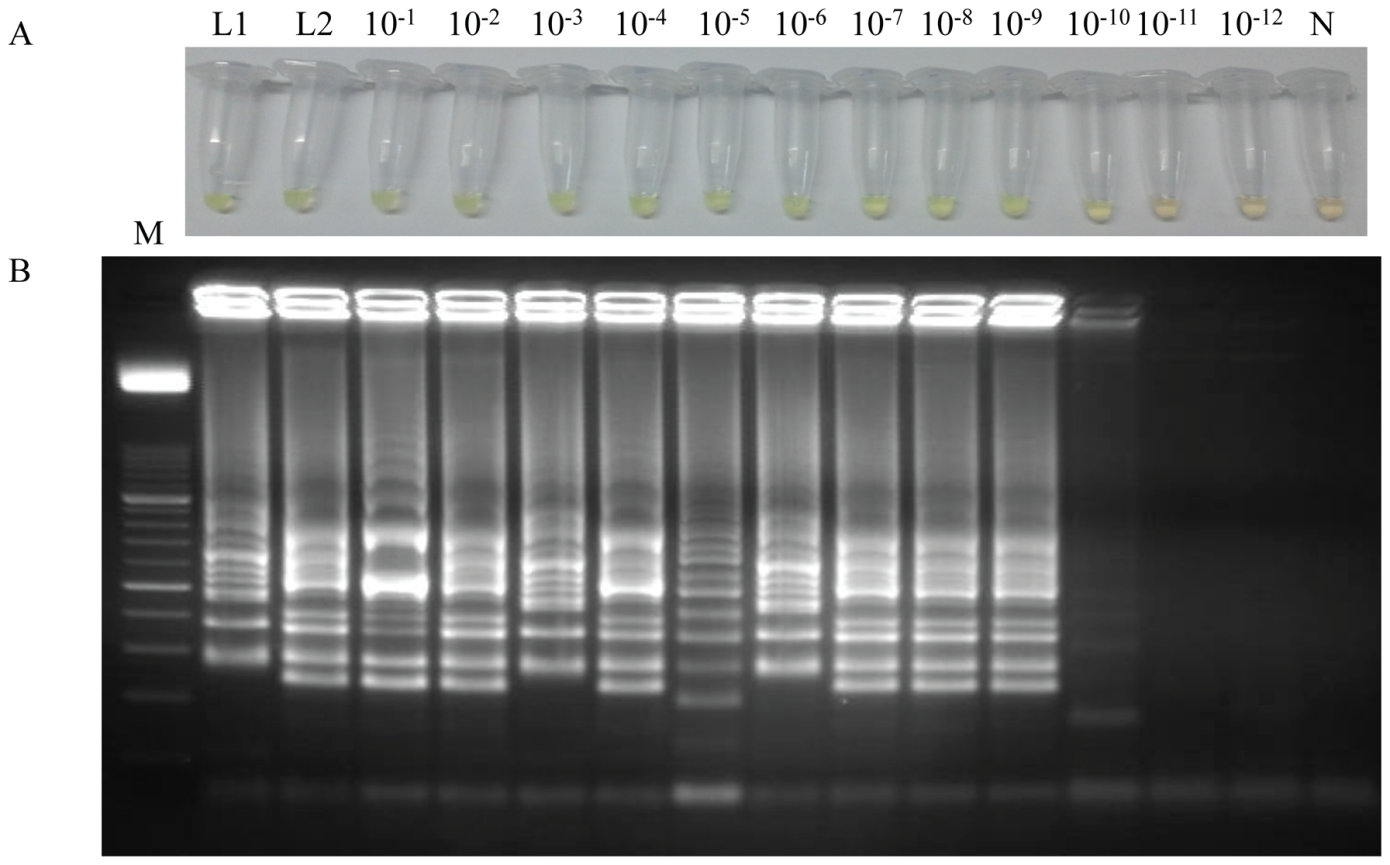
Sensitivity assessment of the LAMP assay for *Loa loa* performed with a heating block using simulated human blood samples spiked with serial dilutions of genomic DNA by the addition of SYBR Green I (A) or by visualization on agarose gel stained with ethidium bromide (B). Lane L1: genomic DNA from *Loa loa* (5 ng) as positive control; lane L2: human blood sample spiked with 5 ng of genomic DNA from *Loa loa*; lanes 10^−1^–10^−12^: human blood samples spiked with 10-fold serially dilutions; lane N: negative control (no DNA template). Lane M: 50 bp DNA ladder (Molecular weight marker XIII, Roche).

The detection limit of LAMP in simulated human blood samples was also examined. The LAMP assay also detected the presence of DNA from *L. loa* down to as little as 0.5 ag when using a conventional heating block to incubate the reaction tubes (Fig. 5).

## Discussion

In our work, a 839 bp species-specific repetitive DNA sequence from *L*. *loa* [10] was selected as a target for a LAMP-based method to detect purified *L. loa* DNA. To determine the specificity of *L. loa* LAMP assay, reactions were conducted for amplification of *L. loa* genomic DNA including heterogeneous DNA from other parasites such us, *Mansonella perstans*, *Brugia pahangi*, *Strongyloides venezuelensis Anisakis simplex*, *Schistosoma mansoni*, *S. haematobium*, *S. intercalatum*, *S. bovis*, *Fasciola hepatica*, *Echinostoma caproni*, *Dicrocoelium dendriticum*, *Hymenolepis diminuta*, *Taenia taeniformis* and *Echinococcus granulosus*. As a result, only *L. loa* DNA was amplified, and no cross-reaction was found when using DNA as a target from other filarial parasites such as *M. perstans*, or even *B. pahangi*, which is a filarial parasite closely related to *L. loa* [22]. Moreover, a conventional PCR was performed with two outer primers to amplify *L. loa* DNA in order to confirm that the LAMP amplified the correct target, thereby ensuring high specificity for target amplification. An important advantage of LAMP is that it allows visual detection of amplification with naked eye inspection either in the form of visual turbidity [17] or visual fluorescence through the addition of fluorescent dyes such as SYBR Green I [23]. In our experiments using LAMP to amplify *L. loa* DNA not only a change in turbidity was clearly visually appreciated but also a visual detection of an orange-to-green color change with SYBR Green I was easily accomplished and always correlated with gel electrophoresis findings.

Regarding the sensitivity of LAMP, it is generally considered that LAMP is highly sensitive compared to PCR methods. In our work, the sensitivity of both conventional PCR using outer primers and LAMP assay was evaluated using *L. loa* genomic DNA which was serially 10-fold diluted. The detection limit of PCR was 0.5 ng of *L. loa* DNA whereas the detection limit of LAMP assay using a thermocycler was 50 fg, showing that LAMP assay is more sensitive than PCR method. Unexpectedly, when LAMP assay was performed in a conventional heating block, the results showed that 0.5 ag of *L. loa* DNA could be detected. Additionally, we obtained identical results when simulated human blood samples spiked with *L. loa* genomic DNA 10-fold serially diluted were used to set up the sensitivity of LAMP. Thus, LAMP assay using thermocycler was 10^4^ times more sensitive than a conventional PCR whereas it was 10^9^ times more sensitive when a conventional heating block was used for amplification. Increasing in sensitivity of LAMP assay when a heating block instead a thermocycler is used to keep both temperature and time for amplification has been observed by us in developing of other LAMP assays in which we are currently working (unpublished data).

It seems logical to consider that using a thermocycler or a heating block to keep both temperature and time for LAMP amplification should not influence on different sensitivities obtained by the technique; however, to use a simple and cheaper incubator could be better to obtain a higher sensitivity. It is known that *Bst* polymerase Large Fragment used in typically LAMP assays for DNA amplification has a 100% activity at 60°C–65°C, but it also has 20% activity up to 70°C. When a quality thermocycler is used for LAMP both temperature and time for DNA amplification can be accurately pre-programmed (*i.e.* 60 min at 63°C; 5 min at 80°C) and the cycler raises and lowers temperature of the block to achieve fast temperature changes in a scheduled time. However, when an economical heating block is used for LAMP, time programming and fast temperature changes are not possible. Once the reaction tubes have been incubated for 60 min at 63°C, a manually change to increase temperature to achieve heat-inactivation of the *Bst* polymerase (greater than 70°C; typically 80°C for 5–10 min) is required. That temperature increase is commonly slow as the block is heating up gradually and may take some time to get heat-inactivation of the *Bst polymerase*, thus enabling the enzyme to work for longer at a range of temperatures from started 63°C up to 70°C; in this timeframe the *Bst polymerase* has certain activity before it becomes heat-inactivated and, therefore, still yielding amplification.

The detection limit obtained with our primer set designed for LAMP to detect *L. loa* genomic DNA (0.5 ag) was found to be higher than one obtained by real-time PCR (0.1 pg) described by Fink *et al* (15) and also than those obtained by other LAMP assays designed for DNA detection from other pathogenic filarial worms. Thus, a LAMP diagnostic assay for *brugian filariasis* detected 1 pg of total genomic DNA purified from *Brugia malyi* worms (9 times less sensitive) [20]; other LAMP assays designed to detect DNA from *Wuchereria bancrofti* [21] and *Dirofilaria immitis* [19] have reported a detection limit of 0.1 pg of filarial DNA (6 times less sensitive). It has been estimated that a single microfilaria contains approximately an amount of 100 pg of DNA [24], [25], [26]. Using our LAMP assay it is possible to detect as little as 0.5 ag of total genomic DNA purified from *L. loa* worm which is equivalent to 1/1.000.000^th^ of a microfilaria.

We are aware that our LAMP assay has not been tested with clinical samples, but our data obtained in simulated human blood samples indicate that the LAMP assay designed is sensitive enough to detect *L. loa* at a very low level, and thus the use of this technique may have important clinical applications. It has been reported that the sensitivity of LAMP was less affected by the various components of the clinical samples than was PCR and, moreover, DNA purification from samples could be omitted [27]. In this context, and considering the high sensitivity obtained in detecting *L. loa* DNA in simulated human blood samples, our assay could be potentially use in screening clinical samples either with extracted DNA or just with boiled whole blood.

In conclusion, the results of the current study demonstrated that the established LAMP assay is cost-effective, specific and very highly sensitive technique for detection of *L. loa* DNA. Although further research for evaluation of the method for the application in clinical samples is required, the method is potentially adaptable for field diagnosis and disease surveillance in loiasis-endemic areas.

## References

[1] Boussinesq M (2006) Loiasis. Ann Trop Med Parasitol 100: 715–731. 17227650 10.1179/136485906X112194

[2] Addiss DG, Rheingans R, Twum-Danso NA, Richards FO (2003) A Framework for Decision-Making for Mass Distribution of Mectizan (R) in Areas Endemic for *Loa loa* . Filaria J2 (Suppl 1)S9. 10.1186/1475-2883-2-S1-S9 PMC2147661 14975066

[3] Pion DS, Gardon J, Kamgno J, Gardon-Wendel N, Chippaux JP, et al (2004) Structure of the microfilarial reservoir of *Loa loa* in the human host and its implications for monitoring the programmes of Community-Directed Treatment with Ivermectin carried out in Africa. Parasitology 129: 613–626. 15552406 10.1017/s0031182004005694

[4] Twum-Danso NA (2003) *Loa loa* encephalopathy temporally related to ivermectin administration reported from onchocerciasis mass treatment programs from 1989 to 2001: implications for the future. Filaria J2 (Suppl 1)S7. 10.1186/1475-2883-2-S1-S7 PMC2147656 14975064

[5] Mackenzie C, Geary T, Prichard R, Boussinesq M (2007) Where next with *Loa loa* encephalopathy? Data are badly needed. Trends Parasitol 23: 237–238. 17459773 10.1016/j.pt.2007.04.007

[6] Lal RB, Ottesen EA (1988) Enhanced diagnostic specificity in human filariasis by IgG4 antibody assessment. J Infect Dis 158: 1034–1037. 2460565 10.1093/infdis/158.5.1034

[7] Akue JP, Egwang TG, Devaney E (1994) High levels of parasitespecific IgG4 in the absence of microfilaremia in *Loa loa* infection. Trop Med Parasitol 45: 246–248. 7899797

[8] Klion AD, Vijaykumar A, Oei T, Martin B, Nutman TB (2003) Serum immunoglobulin G4 antibodies to the recombinant antigen, Ll-SXP-1, are highly specific for *Loa loa* infection. J Infect Dis 187: 128–133. 12508156 10.1086/345873

[9] Burbelo PD, Ramanathan R, Klion AD, Iadarola MJ, Nutman TB (2008) Rapid, novel, specific, high-throughput assay for diagnosis of *Loa loa* infection. J Clin Microbiol 46: 2298–2304. 18508942 10.1128/JCM.00490-08 PMC2446928

[10] Klion AD, Raghavan N, Brindley PJ, Nutman TB (1991) Cloning and characterization of a species-specific repetitive DNA sequence from *Loa loa* . Mol Biochem Parasitol 45: 297–305. 2038361 10.1016/0166-6851(91)90098-q

[11] Toure FS, Bain O, Nerrienet E, Millet P, Wahl G, et al (1997) Detection of *Loa loa*-specific DNA in blood from occult-infected individuals. Exp Parasitol 86: 163–170. 9225766 10.1006/expr.1997.4168

[12] Toure FS, Kassambara L, Williams T, Millet P, Bain O, et al (1998) Human occult loiasis: improvement in diagnostic sensitivity by the use of a nested polymerase chain reaction. Am J Trop Med Hyg 59: 144–149. 9684643 10.4269/ajtmh.1998.59.144

[13] Toure FS, Mavoungou E, Kassambara L, Williams T, Wahl G, et al (1998) Human occult loiasis: field evaluation of a nested polymerase chain reaction assay for the detection of occult infection. Trop Med Int Health 3: 505–511. 9657514 10.1046/j.1365-3156.1998.00260.x

[14] Jiménez M, González LM, Carranza C, Bailo B, Perez-Ayala A, et al (2011) Detection and discrimination of *Loa loa*, *Mansonella perstans* and *Wuchereria bancrofti* by PCR-RFLP and nested-PCR of ribosomal DNA ITS1 region. Exp Parasitol 127: 282–286. 20599994 10.1016/j.exppara.2010.06.019

[15] Fink DL, Kamgno J, Nutman TB (2011) Rapid molecular assays for specific detection and quantitation of *Loa loa* microfilaremia. PLoS Negl Trop Dis. Aug 5(8): e1299 10.1371/journal.pntd.0001299 PMC3164211 21912716

[16] Notomi T, Okayama H, Masubuchi H (2000) Loop-mediated isothermal amplification of DNA. Nucleic Acids Res 28: E63. 10871386 10.1093/nar/28.12.e63 PMC102748

[17] Mori Y, Nagamine K, Tomita N, Notomi T (2001) Detection of loop-mediated isothermal amplification reaction by turbidity derived from magnesium pyrophosphate formation. Biochem Biophys Res Commun 289: 150–154. 11708792 10.1006/bbrc.2001.5921

[18] Tomita N, Mori Y, Kanda H, Notomi T (2008) Loop-mediated isothermal amplification (LAMP) of gene sequences and simple visual detection of products. Nat Protoc 3: 877–882. 18451795 10.1038/nprot.2008.57

[19] Aonuma H, Yoshimura A, Perera N, Shinzawa N, Bando H, et al (2009) Loop-mediated isothermal amplification applied to filarial parasites detection in the mosquito vectors: *Dirofilaria immitis* as a study model. Parasit Vectors 2: 15 10.1186/1756-3305-2-15 19284882 PMC2670822

[20] Poole CB, Tanner NA, Zhang Y, Evans TC Jr, Carlow CK (2012) Diagnosis of brugian filariasis by loop-mediated isothermal amplification. PLoS Negl Trop Dis 6(12): e1948 10.1371/journal.pntd.0001948 23272258 PMC3521703

[21] Takagi H, Itoh M, Kasai S, Yahathugoda TC, Weerasooriya MV, et al (2011) Development of loop-mediated isothermal amplification method for detecting *Wuchereria bancrofti* DNA in human blood and vector mosquitoes. Parasitol Int 60: 493–497. 21930238 10.1016/j.parint.2011.08.018

[22] Fong MY, Asha T, Azdayanti M, Yee LL, Sinnadurai S, et al (2008) Inferring the phylogenetic position of *Brugia pahangi* using 18S ribosomal RNA (18S rRNA) gene sequence. *Trop Biomed* 25: 87–92. 18600209

[23] Parida M, Sannarangaiah S, Dash PK, Rao PV, Morita K (2008) Loop mediated isothermal amplification (LAMP): a new generation of innovative gene amplification technique; perspectives in clinical diagnosis of infectious diseases. Rev Med Virol. 18: 407–421. 10.1002/rmv.593 PMC7169140 18716992

[24] Lizotte MR, Supali T, Partono F, Williams SA (1994) A polymerase chain reaction assay for the detection of *Brugia malayi* in blood. Am J Trop Med Hyg 51: 314–321. 7943550 10.4269/ajtmh.1994.51.314

[25] Rao RU, Weil GJ, Fischer K, Supali T, Fischer P (2006) Detection of *Brugia* parasite DNA in human blood by real-time PCR. J Clin Microbiol 44: 3887–3893. 16957038 10.1128/JCM.00969-06 PMC1698366

[26] Rao RU, Atkinson LJ, Ramzy RM, Helmy H, Farid HA, et al (2006) A real time PCR-based assay for detection of *Wuchereria bancrofti* DNA in blood and mosquitoes. Am J Trop Med Hyg 74: 826–832. 16687688 PMC2196401

[27] Kaneko H, Kawana T, Fukushima E, Suzutani T (2007) Tolerance of loop-mediated isothermal amplification to a culture medium and biological substances. J Biochem Biophys Methods 70: 499–501. 17011631 10.1016/j.jbbm.2006.08.008

